# Supplementary material for: A Candidate DNA Vaccine Encoding the Native SARS-CoV-2 Spike Protein Induces Anti-Subdomain 1 Antibodies
Source: Vaccines (Basel). 2023 Sep 3;11(9):1451. doi: 10.3390/vaccines11091451 (PMC10535225; doi:10.3390/vaccines11091451)
Supplement: Supplementary file 1 [file vaccines-11-01451-s001.zip › vaccines-2548644-supplementary.pdf]

# Supplementary Material

## Supplementary Figures

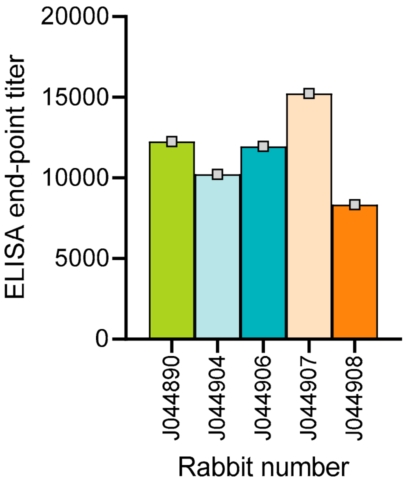


**Supplementary Figure S1. ELISA IgG titers against SARS-CoV-2 D614G spike ectodomain for the complete group of rabbits immunized intramuscularly with a candidate DNA vaccine encoding the index strain spike protein of SARS-CoV-2.** Individual animal data presented here are from the original study describing the pre-clinical evaluation of the DNA vaccine [1]. Animals selected for further analysis on the microarray are J044890, J044906, and J044908.

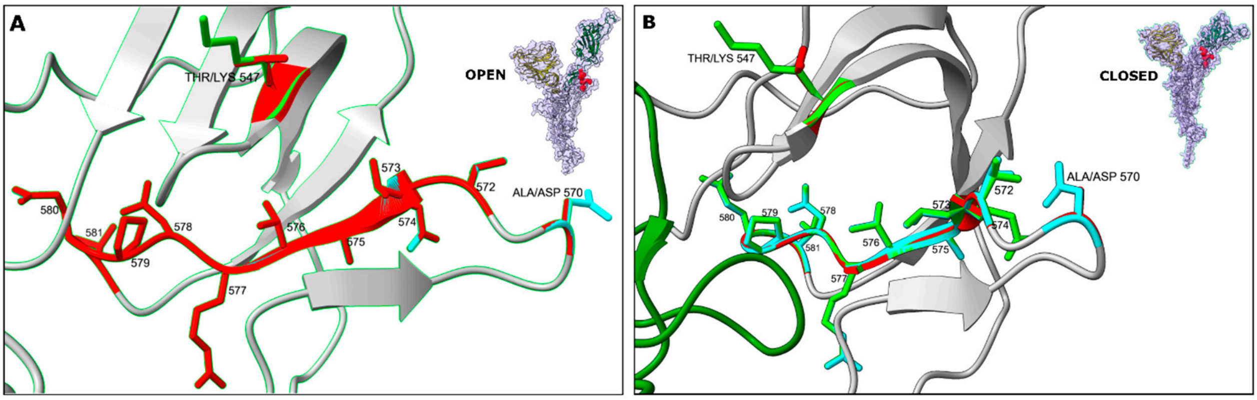


**Supplementary Figure S2. Structural Conservation of the SD1 Epitope in BA.1 and Alpha lineages.** Homology models of SARS-CoV-2 spike monomers either for index strain (red) or incorporating nonsynonymous SD1 mutations in the Omicron BA.1 (green) and Alpha (cyan) lineages upstream of the HomologySD1 epitope were generated with SWISS-MODEL [2] using an appropriate template for each of the open (**A**, PDB ID: 7T9K) and closed conformations (**B**, PDB ID: 7KRQ) and were aligned using the Needleman-Wunsch algorithm [3]. Residues along the SD1 epitope are numbered to show amino acid position and upstream mutations Thr457Lys for the Omicron BA.1 lineage and Ala570Asp for the Alpha lineage are shown. All structural manipulations were performed with UCSF Chimera X (https://www.rbvi.ucsf.edu/chimerax).

**Supplementary References**

1. Lassaunière R, Polacek C, Gram GJ, Frische A, Tingstedt JL, Krüger M, Dorner BG, Cook A, Brown R, Orekov T, et al. Preclinical evaluation of a candidate naked plasmid DNA vaccine against SARS-CoV-2. npj Vaccines (2021) 6:1–13. doi: 10.1038/s41541-021-00419-z

2. Waterhouse A, Bertoni M, Bienert S, Studer G, Tauriello G, Gumienny R, Heer FT, de Beer TAP, Rempfer C, Bordoli L, et al. SWISS-MODEL: homology modelling of protein structures and complexes. Nucleic Acids Res (2018) 46:W296–W303. doi: 10.1093/nar/gky427

3. Needleman SB, Wunsch CD. A general method applicable to the search for similarities in the amino acid sequence of two proteins. J Mol Biol (1970) 48:443–453. doi: https://doi.org/10.1016/0022-2836(70)90057-4
